# Dopaminergic and Prefrontal Basis of Learning from Sensory Confidence and Reward Value

**DOI:** 10.1016/j.neuron.2019.11.018

**Published:** 2020-02-19

**Authors:** Armin Lak, Michael Okun, Morgane M. Moss, Harsha Gurnani, Karolina Farrell, Miles J. Wells, Charu Bai Reddy, Adam Kepecs, Kenneth D. Harris, Matteo Carandini

**Affiliations:** 1 UCL Institute of Ophthalmology, University College London, London WC1E 6BT, UK; 2 UCL Queen Square Institute of Neurology, University College London, London WC1E 6BT, UK; 3 Centre for Systems Neuroscience, University of Leicester, Leicester LE1 7RH, UK; 4 Cold Spring Harbor Laboratory, 1 Bungtown Road, Cold Spring Harbor, NY 11724, USA

**Keywords:** Reinforcement learning, Decision confidence, Psychophysics, Mice, Electrophysiology, Calcium imaging, Optogenetics

## Abstract

Deciding between stimuli requires combining their learned value with one’s sensory confidence. We trained mice in a visual task that probes this combination. Mouse choices reflected not only present confidence and past rewards but also past confidence. Their behavior conformed to a model that combines signal detection with reinforcement learning. In the model, the predicted value of the chosen option is the product of sensory confidence and learned value. We found precise correlates of this variable in the pre-outcome activity of midbrain dopamine neurons and of medial prefrontal cortical neurons. However, only the latter played a causal role: inactivating medial prefrontal cortex before outcome strengthened learning from the outcome. Dopamine neurons played a causal role only after outcome, when they encoded reward prediction errors graded by confidence, influencing subsequent choices. These results reveal neural signals that combine reward value with sensory confidence and guide subsequent learning.

## Highlights

• Mouse choices depend on present confidence, learned rewards, and past confidence
• Choices constrain a model that predicts activity in prefrontal and dopamine neurons
• Learning relies on prefrontal signals encoding predicted value
• Learning relies on dopamine signals encoding prediction error but not predicted value

## Teaser

Lak et al. model the choices made by mice in a visual task with biased rewards and establish neural correlates of the model’s variables, revealing how choices and learning depend on sensory confidence and reward value.

## Introduction

Making decisions often requires combining present sensory evidence with previous reward values and learning from the resulting outcome. It is not known, however, how the brain performs these computations. Studies of perceptual decisions established that observers carry estimates of sensory confidence, i.e., the probability that a percept is correct (Gold and Shadlen, 2007, Kepecs et al., 2008, Kiani and Shadlen, 2009). Studies of reward learning revealed how decisions are informed by past rewards and modeled this process as reinforcement learning (Daw and Doya, 2006, Lee et al., 2012, Samejima et al., 2005, Schultz, 2015, Sutton and Barto, 1998). Animals and humans efficiently combine these computations (Fan et al., 2018, Feng et al., 2009, Hirokawa et al., 2017, Whiteley and Sahani, 2008). However, it is not known what neuronal signals underlie this combination.

A candidate substrate for this combination are the dopamine neurons in ventral tegmental area (VTA). These neurons encode predicted value prior to outcome and reward prediction error after outcome (Bayer and Glimcher, 2005, Cohen et al., 2012, Schultz et al., 1997). They play a causal role in learning from past rewards (Hamid et al., 2016, Kim et al., 2012, Parker et al., 2016, Stauffer et al., 2016, Steinberg et al., 2013, Tsai et al., 2009), and their responses are graded not only by reward value but also by sensory confidence (Lak et al., 2017).

Another candidate for signals combining sensory confidence and past rewards is the medial prefrontal cortex (mPFC). This region sends and receives projections from midbrain dopamine neurons (Beier et al., 2015, Carr and Sesack, 2000, Morales and Margolis, 2017). Neurons in mPFC encode future rewards as inferred from past outcomes (Moorman and Aston-Jones, 2015, Otis et al., 2017, Pinto and Dan, 2015, Pratt and Mizumori, 2001, Rushworth et al., 2011). Lesion or inactivation of mPFC renders animals insensitive to reward value (Corbit and Balleine, 2003, Killcross and Coutureau, 2003, Ostlund and Balleine, 2005, Passecker et al., 2019) and might impair sensory detection (Le Merre et al., 2018).

It is not known whether neurons in these regions compute predicted value by combining sensory confidence and learned value, in a manner that quantitatively accounts for the observed choices. When choosing between stimuli, the appropriate way to compute predicted value is to multiply one’s confidence in the accuracy of the choice with the learned value of that choice (Dayan and Daw, 2008, Lak et al., 2017). It is not known whether the activities of mPFC neurons and VTA dopamine neurons reflect this computation and whether they play similar and causal roles in shaping decisions.

To address these questions, we developed a decision task for mice that requires combining past rewards with present sensory evidence. We devised a simple behavioral model that describes their choices and correctly predicts a seemingly paradoxical effect: that sensory confidence in one trial affects choices in the next trial. The model makes trial-by-trial estimates of predicted value and prediction error, both of which depend on confidence and past rewards. We found precise correlates of predicted value in the pre-outcome activity of mPFC neurons and of VTA dopamine neurons and precise correlates of prediction error in the post-outcome activity of dopamine neurons. Optogenetic manipulations revealed that learning depends on pre-outcome activity of mPFC neurons, but not dopamine neurons, and on the post-outcome activity of dopamine neurons, but not mPFC neurons. These results reveal how frontal and dopamine circuits guide learning under sensory and value uncertainty.

## Results

We begin by describing the behavioral task and the model that fits the observed choices. We then establish correlates for the model’s internal variables in mPFC neurons and in VTA dopamine neurons and demonstrate their specific, causal roles in learning.

### Behavioral Signatures of Learning Guided by Sensory Confidence and Reward Value

**Figure 1:**
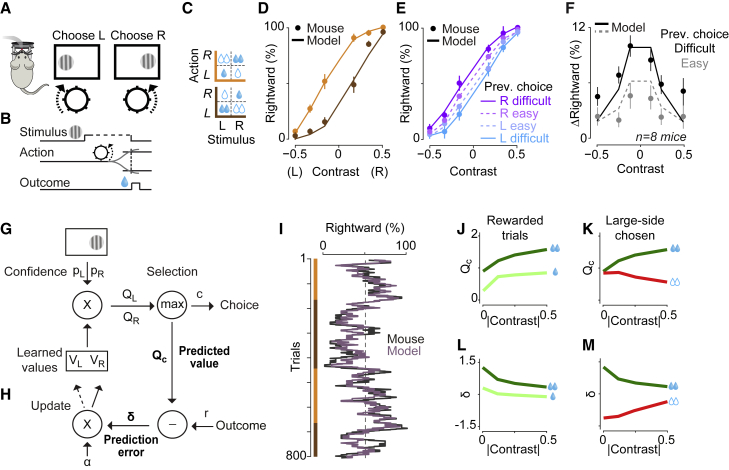
Behavioral and Computational Signatures of Decisions Guided by Reward Value and Sensory Confidence (A and B) Schematic of the 2-alternative visual task. After the mouse kept the wheel still for at least 0.5 s, a sinusoidal grating stimulus of varying contrast appeared on either the left or right monitor, together with a brief tone (0.1 s, 12 kHz) indicating that the trial had started. The mouse reported the choice by steering the wheel located underneath its forepaws. (C) Rewards for correct choices were higher on the right side (orange) or on the left side (brown), with the more-rewarded side switching in blocks of 50–350 trials. (D) Choices of an example mouse in blocks with large reward on right (orange) or left (brown). Curves in this and subsequent panels are predictions of the behavioral model in (G) and (H), and error bars show SE across trials. See Figures S1B–S1D for similar results from all mice, for learning curves and for reaction times. (E) Choices of the same mouse depend on whether the previous rewarded trials were difficult (low contrast) or easy (high contrast). (F) Average change in the proportion of rightward choices after correct decisions in difficult (black) and easy (gray) choices, averaged across mice. (G and H) Behavioral model of choice (G) and learning (H). (I) Running average of probability of choosing right, in a session containing four blocks (orange versus brown). Black: mouse behavior. Light purple: model predictions. (J) Averaged estimates of $Q_{C}$ as a function of absolute contrast (i.e., regardless of side), for correct decisions toward the large-reward side (dark green) and correct decisions toward the small-reward side (light green). (K) Averaged estimates of $Q_{C}$ for correct decisions (dark green) versus incorrect decisions (red), both made toward the large-reward side. See Figure S1J for errors toward small-reward side. (L and M) Similar to (J) and (K) but for reward prediction error δ.

To study decisions guided by sensory signals and reward values, we developed a task for head-fixed mice (Figures 1A–1C). We presented a grating on the left or right side and the mouse indicated the grating’s position by steering a wheel with its forepaws (Figure 1A) receiving water for correct responses (Figure 1B) or a noise sound for incorrect ones (Burgess et al., 2017). To manipulate sensory confidence, we changed the visual contrast of the grating randomly across trials. To manipulate value, we changed the size of rewards for correct left and right choices, with one side receiving twice as much water (2.4 versus 1.2 μL); the more-rewarded side switched without warning in blocks of 50–350 trials and was not otherwise cued (Figure 1C).

Mice mastered this task, efficiently combining current sensory evidence with past rewards (Figure 1D). In this task, high-contrast stimuli are unambiguous and should always be chosen, because choosing the other side would give no reward. Conversely, in low-contrast trials, decisions should favor the side paired with larger reward, as can be derived mathematically (Whiteley and Sahani, 2008) and with simulations (Figure S1A). Mice mastered the task: their psychometric curves shifted sideways between blocks (Figure 1D; Figure S1B), so that reward value predominantly affected decisions for low-contrast stimuli (p < 10^−10^, 1-way ANOVA).

Mouse choices, however, also depended on a seemingly irrelevant factor: the sensory confidence in the previous trial (Figures 1E and 1F). After a correct trial, the psychometric curve shifted toward the chosen side if that trial had been difficult (low contrast) but not if it had been easy (high contrast, Figures 1E and 1F; difficult: p = 0.01, easy: p = 0.56, 1-way ANOVA). These results could not be explained by the presence or absence of rewards (only rewarded trials were included in the analysis), by a win-stay strategy, or by the block structure of the task (the analysis was performed within blocks). Moreover, this effect was not due to the correlation of choices over trials that would be expected from the side bias, Figure 1F; STAR Methods). Indeed, the effect was also present in purely visual decisions, i.e., without manipulation of reward value (Figures S1E and S1F; difficult: p = 0.01, easy: p = 0.12, 1-way ANOVA).

Mouse decisions in this task, therefore, reflect computations that are in some aspects beneficial and in others detrimental for maximizing rewards. The shift in the psychometric curve that follows changes in reward size (Figure 1D) is beneficial. Conversely, the dependence of psychometric curve on past sensory confidence (Figure 1E) is detrimental, because the stimuli were presented in random order. This shift indicates that sensory confidence influences the signals that guide learning. We will see next that it provides a fundamental constraint to a model of behavior.

### A Model for Decisions and Learning Based on Confidence and Reward

To describe mouse behavior and make testable predictions about its neural basis, we used a model that combines signal detection with reinforcement learning (Figures 1G and 1H) (Lak et al., 2017). In the model, the visual system estimates the probabilities $p_{L}$ and $p_{R}$ that the stimulus is on the left or right side. These estimates are noisy: they vary across trials even if these trials involve the same stimulus contrast. Multiplying these quantities with the learned values of the two actions, $V_{L}$ and $V_{R}$, provides the expected values of the two possible choices: $Q_{L}=p_{L}V_{L}$ and $Q_{R}=p_{R}V_{R}$. The higher of these two determines the choice C (either L or R), its sensory confidence $p_{C}$, and its predicted value $Q_{C}=$ $p_{C}V_{C}$. Following the outcome, the model learns by updating the value of the chosen action by $V_{C}\leftarrow V_{C}+\alpha \delta$, where α is a learning rate, and $\delta =r-Q_{C}$ is the reward prediction error, i.e., the difference between available and predicted reward.

This model accounted quantitatively for the animals’ decisions (Figures 1D–1F and 1I). It fitted the shift in psychometric curves due to reward size (Figure 1D, curves), it predicted trial-by-trial decisions (Figure 1I, purple trace), and it captured the time course of learning after block changes (Figure S1C). The model also accounted for the effect of past decision confidence on subsequent choices (Figure 1F, curves; Figures S1E and S1F, curves). Indeed, learning depends on prediction errors that are larger when predicted value $Q_{C}$ is smaller, as is the case at low contrast (where sensory confidence $p_{C}$ is low, because ${\text{p}}_{\text{L}}\approx {\text{p}}_{\text{R}}\approx 0.5$). Cross-validation confirmed the necessity of each model parameter; the full model (with all parameters) provided the best fit in 8 of 10 mice (Figures S1H and S1I).

Conversely, an alternative model that fully leverages the structure of the task did not provide adequate fits (Figures S1L–S1P). A “model-based” observer that knows that only two reward sizes are available and that they occasionally switch side would only need to monitor whether a switch has occurred (Figure S1L). This observer’s choices, however, would not depend on sensory confidence in previous trials (Figures S1M–S1P).

Our behavioral model makes testable predictions for a key internal variable, the predicted value of choice, $Q_{C}$ (Figures 1J and 1K). This variable is computed before the outcome but is on average different for choices that will result to be correct or incorrect. For correct choices, predicted value $Q_{C}$ increases with stimulus contrast and is higher when stimuli appear on the large-reward side (Figure 1J). For incorrect choices, predicted value tends to be lower, because the sensory confidence $p_{C}$ tends to be lower (Figure 1K; Figure S1J).

Similarly, the model makes testable predictions for the reward prediction error, δ (Figures 1L and 1M). This quantity is larger following a larger reward and decreases with stimulus contrast in correct trials (Figure 1L) but not in error trials, again reflecting the difference in sensory confidence across these trials (Figure 1M; Figure S1K).

### Medial Prefrontal Neurons Encode Confidence-Dependent Predicted Value

**Figure 2:**
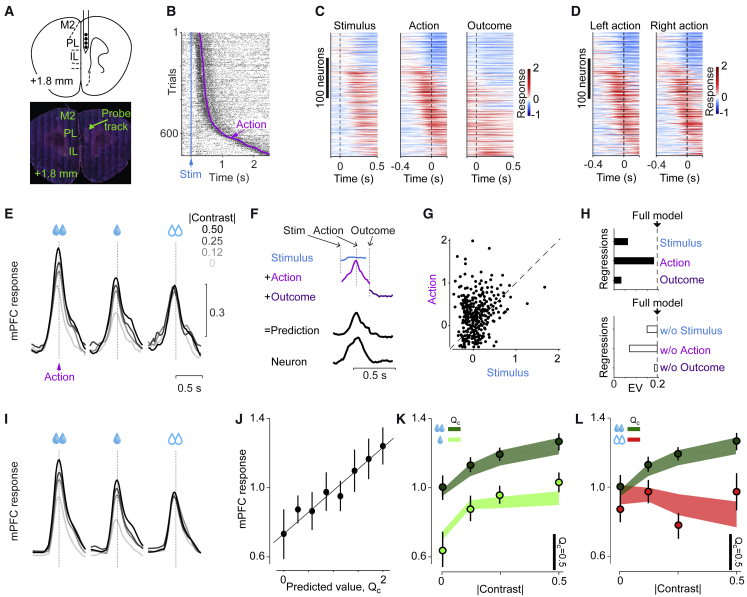
Medial Prefrontal Neurons Encode Confidence-Dependent Predicted Value (A) Histological image showing the high-density silicon probe track in mPFC. (B) Raster plot showing spikes of an example mPFC neuron, aligned to the stimulus onset (blue line) with trials sorted by action onset (purple dots). (C) Responses of all task-responsive neurons (n = 316), aligned to the time of stimulus, action, or outcome, sorted according to the time of maximum response in the middle panel. Responses were *Z* scored and averaged over all stimulus contrasts and possible outcomes. (D) Same as the middle panel of (C) for trials with maximum stimulus contrast with left or right actions. (E) Mean population activity (n = 316 neurons) triggered on action onset for correct choices toward the large-reward side (left), correct choices toward the small-reward side (middle), and incorrect choices toward the large-reward side (right). Responses for incorrect choices toward the small-reward side were smaller (p = 0.015, signed-rank test, data not shown) but such trials were rare. See Figure S2A for responses shown separately for neurons activated or suppressed at the time of the action. See Figure S2B for population activity triggered on outcome onset. (F) The regression analysis estimates a temporal profile for each task event, which in each trial is aligned to the event onset time and scaled by a coefficient. The results are summed to produce predicted traces. (G) The size of action and stimulus profiles for the full regression. Each dot presents one neuron (n = 316). (H) Top: cross-validated explained variance (EV) averaged across neurons (n = 316) for the full regression (dotted line) and for regressions each including only one type of event (bars). Bottom: variance explained by full regression (dotted line) and regressions each excluding one of the events (bars). (I) Predictions of the regression only including action events triggered on action onset, as a function of stimulus contrast and trial type. (J) Average action responses (estimated by regression on mPFC activity) as a function of trial-by-trial decision value $Q_{C}$ (estimated from the behavioral model). Trial-by-trial variations in action-related activity (estimated from the regression) better correlated with $Q_{C}$ in neurons with positive profile, i.e., activated neurons, compared to neurons with negative profile, i.e., suppressed neurons (Figure S2E, p = 0.011, signed-rank test), consistent with results from averaging across neuronal responses (Figure S2A). (K) Average action responses in correct trials as a function of stimulus contrast and reward size. Circles: mean; error bars: SE across neurons; shaded regions: model estimate of $Q_{C}$. (L) Same as (K) but for correct and error trials to the large-reward side. In (J)–(L), only neurons with significant action profile were included (241/316 neurons). See Figures S2F and S2G for responses of remaining neurons.

Seeking to identify neural correlates of the predicted value of choice $Q_{C}$, we recorded the activity of neurons in mPFC (Figure 2A). We used high-density silicon probes to record from 1,566 neurons in the prelimbic area (PL) of 6 mice. Of these, 316 neurons were significantly modulated by at least one task event (signed-rank test on responses prior and after each task event, p < 0.01). A typical neuron fired slightly more following the stimulus and markedly more at the time of action, i.e., the onset of wheel movement (Figure 2B). Among the 316 task-responsive neurons, most were modulated by action onset (78% of the neurons, p < 0.01, signed-rank test) and fewer by stimulus appearance (24%) or outcome delivery (19%, Figure 2C). Most neurons (54%) increased their firing prior to actions, while others (24%) decreased their firing (Figure 2C; p < 0.01, signed-rank test, n = 130–1,080 trials depending on session).

Around the time of the action, several aspects of mPFC activity were consistent with a signal encoding predicted value (Figures 2D and 2E). First, most mPFC neurons (95%) did not respond differently for the left and right actions (p < 0.01, signed-rank test, Figure 2D). Second, mPFC activity depended both on stimulus contrast and on upcoming outcome (Figure 2E). In correct trials (Figure 2E, green), activity around action onset (−200 to 50 ms window) was higher when stimuli had higher contrast (p = 10^−6^, 1-way ANOVA) and were associated with larger rewards (p = 0.009, signed-rank test). In error trials (red), which were most common in decisions toward the large-reward side, mPFC activity was lower than in correct trials (Figure 2E, *red p =* 10^−8^, signed-rank test) and was not significantly modulated by contrast (p = 0.24, 1-way ANOVA). The effects of stimulus contrast, reward size, and correct/error were specific to neurons activated by action and largely absent in neurons suppressed by action (Figure S2A).

To quantify the trial-by-trial activity of mPFC neurons, we focused on their dominant responses, which occurred before the outcome, at the time of action (Figures 2F–2I). We used regression to express each neuron’s activity as a sum of responses related to stimulus, action, and reward, with the magnitude of these responses, but not their temporal profile, allowed to vary between trials (Figure 2F; STAR Methods). Responses related to action were larger than responses related to stimuli and outcomes (Figure 2G, p = 0.0001, signed-rank test). In most neurons (241/316), activity could be explained by action responses alone (Figures 2H and 2I; Figures S2C–S2E). Here, we focus on these neurons (see Figures S2F and S2G for the properties of the remaining neurons).

The pre-outcome activity of many mPFC neurons reflected the predicted value of the choice $\;Q_{C}$ (Figures 2J–2L). Trial-by-trial variations in action-related activity correlated strongly with $Q_{C}$ (Figure 2J, R^2^ = 0.88, p = 10^−4^, linear regression). Similar to $Q_{C}$, mPFC activity increased with the size of the pending reward. Moreover, it increased with stimulus contrast (Figure 2K, dark green and light green), and it did so only for correct decisions (Figure 2L). Trial-by-trial variations in mPFC activity correlated better with $Q_{C}$ than with measures of movement vigor such as wheel acceleration (Figure S2H; population: p = 0.001, signed-rank test; 54 versus 22 neurons, p < 0.01, partial linear correlation). As we will see, optogenetic manipulations further support this observation. Thus, the pre-outcome activity of a large fraction of mPFC neurons reflects $Q_{C}$, the predicted value of the choice.

### Dopamine Neurons Encode Confidence-Dependent Predicted Value and Prediction Error

**Figure 3:**
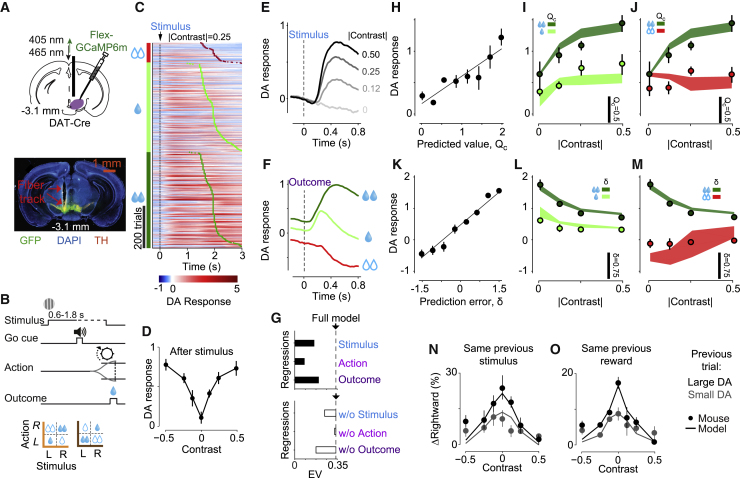
Dopamine Neurons Encode Confidence-Dependent Predicted Value and Prediction Error (A) Top: schematic of fiber photometry in VTA dopamine neurons. Bottom: example histology showing GCaMP expression and the position of implanted fiber above the VTA. (B) Task timeline. To allow sufficient time for GCaMP measurement, decisions could be reported only after an auditory go cue. (C) Trial-by-trial dopamine responses from all sessions of an example animal for trials with |contrast| = 0.25, aligned to stimulus onset (dashed line) and sorted by trial type (left column) and outcome time (red, light-green, and dark-green dots). (D) Dopamine responses of an example animal on correct trials as a function of contrast, for stimuli presented on the left or right side of the monitor. (E) Population dopamine responses (n = 5 mice) aligned to the stimulus. (F) Population dopamine responses aligned to the outcome. (G) Top: cross-validated explained variance (EV) averaged across mice for the full regression (dotted line) and for regressions each including only one type of event (bars). Bottom: EV of full regression (dotted line) and regressions each excluding one of the events (bars). (H) Stimulus responses, estimated from regression, as a function of trial-by-trial decision value $Q_{C}$, estimated by the behavioral model. (I) Average stimulus responses in the correct trials as a function of stimulus contrast and trial type (error bars: SE across animals); shaded regions: model predictions of $Q_{C}$. (J) Same as (I) but for correct and error trials in which the large-reward side was chosen. (K) Outcome responses, estimated from regression, as a function of trial-by-trial prediction error δ, estimated by the behavioral model. (L and M) Same as (I) and (J) for outcome responses and model estimates of δ. (N) Changes in the proportion of rightward choices as a function of dopamine activity to reward in the previous trial (black and gray: larger and smaller than 65 percentile, respectively), computed for each level of sensory stimulus in the previous trial (for left and right blocks separately), and then averaged. (O) Changes in the proportion of rightward choices as a function of dopamine activity to reward in the previous trial, computed for each reward size in the previous trial (for left and right blocks separately), and then averaged.

To examine the activity of VTA dopamine neurons, we measured their responses during the task using fiber photometry of GCaMP6 signals (Figures 3A and 3B). To allow sufficient time to measure Ca^2+^ fluctuations, we modified the task slightly and trained mice to respond after an auditory go cue that followed the visual stimulus (Figure 3B).

Dopamine activity was strongly modulated both at the time of stimulus onset and at the time of outcome (Figures 3C–3G). Following stimulus presentation, dopamine activity increased with the size of pending reward (Figure 3C, p < 0.004 in 5/5 mice, signed-rank test) and with stimulus contrast (Figures 3D and 3E, p < 10^−4^ in 5/5 mice, 1-way ANOVA), largely independently of stimulus side (Figure 3D, p > 0.08 in 5/5 mice, signed-rank test). Dopamine activity was not significantly modulated at the time of go cue or of action (Figure S3, p > 0.1, p > 0.13 in 5/5 mice, signed-rank test; note that the slow time course of GCaMP might hide subtle responses to these events). However, it was markedly increased at the time of outcome, especially after obtaining the larger reward (Figures 3C and 3F, p < 10^−4^ in 5/5 mice, 1-way ANOVA). We used regression to estimate dopamine responses to stimulus presentation, action, and reward on every trial (Figure S3A). Omitting responses to action did not worsen the predictions (Figure 3G; Figures S3B and S3C), so we focused on responses to stimulus and outcome.

Dopamine responses prior to outcome reflected predicted value $Q_{C}$, in a manner similar to mPFC activity, and dopamine responses after the outcome encoded prediction error δ (Figures 3H–3M). At stimulus time, dopamine activity closely followed the behavioral model’s trial-by-trial estimates of predicted value $Q_{C}$ (Figure 3H; population: R^2^ = 0.83, p = 0.001 and R^2^ > 0.57, p < 0.01 in 5/5 mice, linear regression), increasing with pending reward size and stimulus contrast for correct trials (Figure 3I) but not for incorrect trials (Figure 3J). At outcome time, moreover, dopamine activity closely followed the model’s estimates of prediction error δ (Figure 3K; population: R^2^ = 0.97, p = 10^−6^ and R^2^ > 0.88, p < 10^−4^ in 5/5 mice, linear regression). It increased with reward size and depended on the contrast of a stimulus that was no longer on the screen, decreasing with contrast in correct trials (Figure 3L) and not in error trials (Figure 3M).

Consistent with the encoding of prediction error, dopamine responses after outcome correlated with subsequent choices: if a choice was followed by a large dopamine response, mice were more likely to make the same choice in the next trial (Figures 3N and 3O). Larger dopamine response had more influence on next choices, causing a larger shift in the psychometric curves, whether they were due to larger reward size (because the stimulus contrast was identical, Figure 3N; p = 0.0002, 1-way ANOVA) or due to lower sensory confidence (because the reward size was identical, Figure 3O; p = 0.0007, 1-way ANOVA). The behavioral model captured these effects because prediction error δ depends on both sensory confidence and reward value (Figures 3N and 3O, curves).

### Learning Depends on Predicted Value Signaled by Medial Prefrontal Neurons

**Figure 4:**
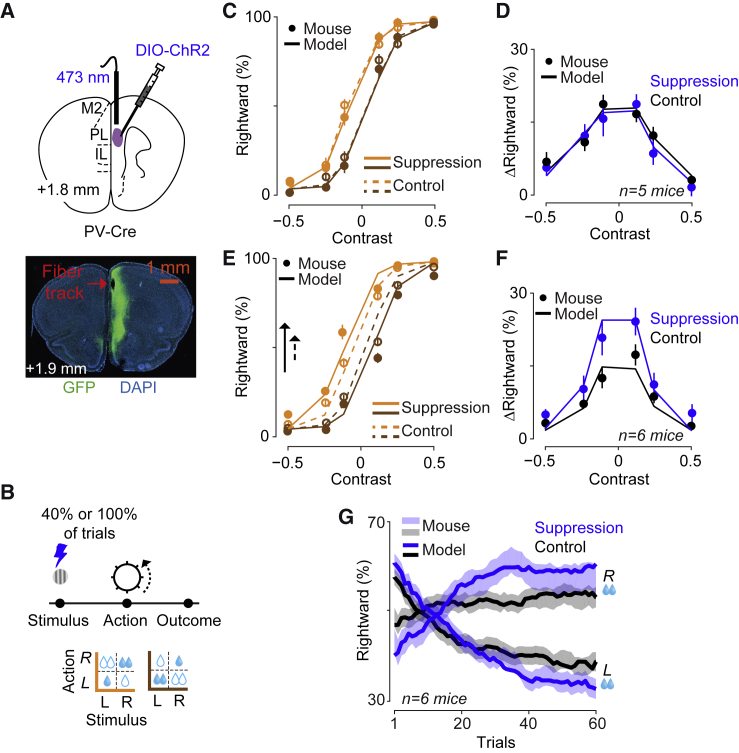
Learning Depends on Predicted Value Signaled by Medial Prefrontal Neurons (A) Top: to suppress mPFC population activity, we optogenetically activated Pvalb neurons by directing brief laser pulses through an optical fiber in the prelimbic area (PL). Bottom: example histology showing ChR2 expression in mPFC and position of implanted fiber above mPFC. (B) Inactivation occurred for 450 ms following stimulus onset in two different forms: in either 40% of randomly selected trials of blocks with reward size manipulation (C and D) or in blocks of trials, forming four possible blocks: with or without suppression; with large reward on the left or the right (E–G). (C) Reducing $Q_{C}$ in the model does not influence ongoing choices. Curves are model predictions for trials with reduced $Q_{C}$ (solid) and control trials (dashed). Consistent with the model prediction, suppressing mPFC neurons did not influence the performance in current trials. See Figure S4B for similar results in a task with no reward manipulation. (D) Effect of mPFC suppression on psychometric shifts in 5 mice. Data points show the difference in the proportion of rightward choices between the L and R blocks of the control and suppression conditions. Curves illustrates average model fits on the data. Error bars show SE across animals. (E) Reducing $Q_{C}$ in the model magnifies psychometric bias due to reward size difference. The arrow indicates the difference in the probability of rightward choice computed from the point curves cross zero contrast in the control (dashed) and in blocks with reduced $Q_{C}$ (solid). Consistent with the model prediction, suppressing mPFC neurons during the task magnified the shifts of psychometric curves due to the reward size difference. The data points show an example animal. (F) Effect of mPFC suppression on psychometric shifts in 6 mice. Curves illustrates average model fits on the data (with reduced $Q_{C}$ relative to control). (G) The effect of mPFC suppression on trial-by-trial learning from the onset of the switches in reward contingencies. The shaded areas indicate data (n = 6 mice) in the control (black) and optogenetic suppression (blue) experiment, and curves are average predictions of the model fitted on the data.

Having established that mPFC signals prior to outcome encode predicted value, $Q_{C}$, we asked whether these signals play a causal role (Figure 4). In our model, $Q_{C}$ is determined only after making the choice C. The model thus predicts that reducing $Q_{C}$ cannot influence the ongoing choice. Rather, reducing $Q_{C}$ should affect learning, thus influencing subsequent choices. We tested these predictions through optogenetic inactivation in mice expressing Channelrhodopsin-2 (Chr2) in *Pvalb*-expressing inhibitory neurons of mPFC (Figures 4A and 4B; Figure S4A) (Guo et al., 2014, Olsen et al., 2012).

Consistent with the first prediction, suppressing mPFC did not disrupt ongoing choices (Figures 4C and 4D). We suppressed mPFC activity in a subset of trials from the stimulus onset for 450 ms and found no significant effect of mPFC suppression on the ongoing choices (p = 0.84, signed-rank test). Similar results were observed in a simpler version of the task where reward sizes are equal and constant; even in this purely visual task, ongoing choices were immune to mPFC inactivation (Figure S4B).

Consistent with the second prediction, suppressing mPFC increased the effect of learning (Figures 4E–4G). In the model, reducing $Q_{C}$ in trials ending with reward would overestimate positive prediction error, $\delta =R-Q_{C}$, magnifying the subsequent shift in psychometric curves. We verified this prediction by suppressing mPFC activity from the stimulus onset in blocks of trials (four possible blocks: with or without mPFC suppression, with large reward on the left or right). Suppressing mPFC significantly increased the shifts in psychometric curves (Figures 4E and 4F, p = 0.01, signed-rank test). The model readily accounted for this effect (Figure 4E, curves) with the simple assumption that inactivation of mPFC subtracts a constant value from $Q_{C}$ (Figure S4C; other model modifications failed to account for the data, see STAR Methods). The model also closely predicted that inactivation of mPFC facilitated the progression of learning after the switch between blocks with different reward contingencies (Figure 4G). These effects of mPFC inactivation were not accompanied by sensory or motor correlates: there were no changes in visual sensitivity (the slope of psychometric curves, p = 0.27, signed-rank test), in reaction time (p = 0.43) or in wheel acceleration (p = 0.53). Also, these effects were seen only when suppressing mPFC activity before outcome: consistent with the weak responses seen in mPFC at the time of outcome, suppressing mPFC at that time in blocks of trials did not influence the choices (Figure S4D, p = 0.96, signed-rank test).

Taken together, these results indicate that mPFC causally encodes predicted value $Q_{C}$. Pre-outcome activity in mPFC is necessary not to make a choice but rather to learn from the outcome and thus shape future behavior.

### Learning Depends on Prediction Error, but Not Predicted Value, Signaled by Dopamine Neurons

**Figure 5:**
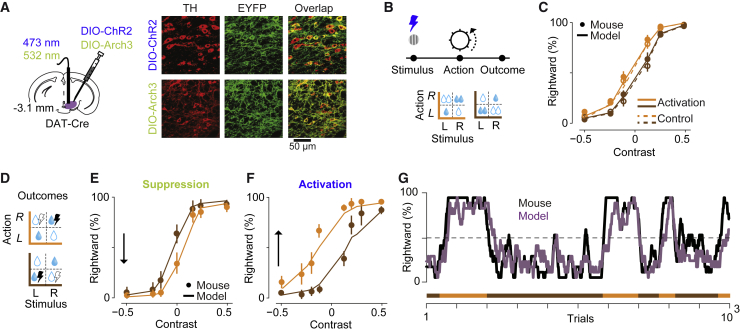
Learning Depends on Prediction Error but Not Predicted Value Signaled by Dopamine Neurons (A) Left: ChR2 or Arch3 were expressed in dopamine neurons and a fiber implanted over VTA. Right: expression of ChR2 or Arch3 in dopamine neurons. (B) In the first experiment, light pulses were delivered at the time of visual stimulus in blocks of trials, forming four possible blocks (with or without inactivation, with large reward on the left or right). (C) Behavior of an example animal in the activation trials (filled circles) and control trials (empty circles). Curves are model fits. Error bars are SE across trials. See Figure S5B for population data and Figures S5C–S5G for similar results in a task without reward manipulation or when activation started before the stimulus onset. (D) Manipulation of dopamine responses at the time of outcome: light pulses were delivered following correct decisions toward one response side, which alternated in blocks of 50–350 trials. (E and F) Model-predicted horizontal psychometric curve shift (curves) accounts for dopamine-induced behavioral changes (points). The arrow indicates the difference across blocks in the probability of rightward choice in trials with zero contrast. The psychometric shifts were independent of the hemisphere manipulated (p = 0.36, 2-way ANOVA). See Figures S5H–S5J for similar results across the population and reaction times. (G) Running average of probability of rightward choice in an example session including 8 blocks (orange and brown). Black: mouse behavior. Purple: model prediction. See Figure S5K for averaged learning curves.

Having observed that dopamine signals encode predicted value $Q_{C}$ before outcome and prediction error δ after outcome, we next investigated their impact on choices (Figure 5). We implanted optical fibers above VTA in mice expressing Archaerhodopsin-3 (Arch3) or Chr2 in midbrain dopamine neurons (Figure 5A; Figure S5A), and we delivered brief laser pulses at the time of stimulus or of water reward.

In striking contrast to the results obtained in prefrontal cortex, manipulating dopamine activity prior to outcome had no effects on choices or learning (Figures 5B and 5C; Figure S5). Activating dopamine neurons at stimulus onset (for 450 ms) did not affect the dependence of psychometric curves on reward size (Figure 5C; p = 0.46, signed-rank test, Figure S5B). It also did not affect the animal’s visual sensitivity (the slope of the psychometric curves, p = 0.67, signed-rank test). We observed similar results when we activated these neurons in a subset of trials in a purely visual task, without reward manipulation, regardless of whether activation coincided with stimulus onset or preceded it (Figures S5C–S5G).

These results reveal a fundamental difference between the signals encoding predicted value in the pre-outcome activity of mPFC neurons and of VTA dopamine neurons. The former plays a causal role in learning, but the latter does not.

In contrast, manipulation of post-outcome dopamine responses drove learning in a manner that was similar to changes in reward size (Figures 5D–5G). We modified our protocol so that the water rewards were equal across sides: the difference between blocks was the side where water was paired with laser pulses (Figure 5D). As expected from signals encoding reward prediction error, suppression and activation of VTA dopamine neurons at the time of outcome had opposite effects on decisions. Suppression shifted decisions away from the side paired with laser pulses, whereas activation shifted the decisions toward that side (Figures 5E and 5F, p < 0.01, 1-way ANOVA; Figures S5H–S5J). Dopamine-dependent psychometric shifts developed over ∼8 trials after block switches (Figure 5G; Figure S5K), compared to ∼12 trials in the experiment with reward size manipulation (Figure S1C). We observed similar psychometric shifts in experiments where we activated dopamine in a random subset of trials rather than in blocks of trials, indicating that dopamine activation in one trial is enough to influence subsequent choices (Figure S5L).

The model’s estimates of reward prediction error δ precisely captured the effects of these manipulations (Figures 5E–5G; Figures S5H–S5N). To model dopamine manipulation, we added to δ a factor that was negative for dopamine suppression and positive for dopamine activation (Figures S5M and S5N). This addition does not lead the model to arbitrarily low or high estimates of value, because as estimates progressively deviate from veridical, they lead to more errors, which correct the estimates toward more reasonable steady-state values (Figure S5O). The model thus captured the behavior of the mice, which did not develop pure biases for one action or the other, but rather shifted their psychometric curves sideways (Figures 5E and 5F) and rapidly reached the steady state (Figure S5K). As predicted by the model, the effect of dopamine manipulation on decisions was graded by the strength of sensory evidence: mice incorporated past dopamine manipulations into their choices only when sensory confidence was weak.

## Discussion

By manipulating both sensory confidence and reward value, we formalized how these two factors shape decisions and guide learning, involving distinct causal roles of mPFC neurons and VTA dopamine neurons. We found that mouse choices reflect not only current sensory evidence and learned rewards but also past decision confidence. Choices were captured by a simple model that infers two key internal variables, one computed pre-outcome, and one computed post-outcome. The first variable, the predicted value of the chosen option, was causally encoded in the activity of mPFC neurons and non-causally reflected in the activity of midbrain dopamine neurons. The second variable, prediction error, was causally encoded in the activity of VTA dopamine neurons. Just as in the behavioral model, both of these signals precisely depended on sensory confidence and on reward history. Also, as in the model, these signals were necessary not for performing the ongoing trial but rather for learning from the trial’s outcome.

We found multiple ways in which mPFC activity precisely conforms to the predicted value of choice, i.e., the product of sensory confidence with reward value. These include (1) the increase of mPFC responses with reward size, (2) the increase of mPFC responses with sensory confidence, (3) the difference in mPFC responses during error choices versus correct choices, and (4) the invariance of mPFC responses with choice direction and stimulus position.

Optogenetic inactivation further revealed the roles of mPFC predicted value signals in learning. Upon making a choice, it is useful to compute its predicted value, so that it can be compared to outcome and drive learning (Sutton and Barto, 1998). Our optogenetic results confirm these predictions: reducing mPFC signals strengthened learning by increasing the shift in psychometric curves and influencing future choices rather than ongoing choices. Thus, our results support the idea that predicted value, just like outcome value, shapes learning driven by prediction error.

Medial prefrontal cortex, however, might be part of a larger network of regions computing predicted value and carrying learning signals. For example, signals encoding economic chosen value have been found in other prefrontal areas (Padoa-Schioppa and Assad, 2006). Signals encoding sensory confidence (one of the two factors that determine predicted value) have been seen in parietal cortex, orbitofrontal cortex, and dorsal pulvinar (Kepecs et al., 2008, Kiani and Shadlen, 2009, Komura et al., 2013), and signals necessary for learning may be observed in orbitofrontal cortex (Miller et al., 2018, Takahashi et al., 2009).

These results may reconcile previous observations about neuronal signals in mPFC. Studies in animals freely moving in a maze or in an operant box argue that PFC neurons can be selective for directional actions (e.g., Feierstein et al., 2006, Spellman et al., 2015, Sul et al., 2010). By contrast, studies in head-fixed mice in non-directional tasks (Pavlovian or go/no go) argue that mPFC neurons respond to stimuli and rewards rather than actions (Le Merre et al., 2018, Otis et al., 2017). Our experiments involved directional movements for reporting choice and showed strong mPFC responses at the time of actions. These responses, however, were not directional, perhaps because the body movements required by our task were smaller than in freely moving animals.

Our manipulations of mPFC activity could be refined by using additional inactivation methods. One limitation of our methods is that to suppress mPFC responses, we activated parvalbumin (PV) inhibitory neurons (Guo et al., 2014, Olsen et al., 2012) that might send long-range projections to the nucleus accumbens (Lee et al., 2014). Our results might thus be due to inactivating nucleus accumbens through those projections. However, we believe this is unlikely for two reasons. First those projections mediate avoidance behavior (Lee et al., 2014), which we did not observe when activating PV populations (Figure S4). Second, it is not clear that PV-Cre mouse lines would efficiently label these projections (Lee et al., 2014). Another limitation of our methods is that we manipulated mPFC with laser pulses at 25 Hz, which could have led to beta frequency oscillations, and might have functional consequences (Buschman and Miller, 2007, van de Vijver et al., 2011). We think this is unlikely, because the effects of optogenetic manipulation of mPFC have been found to be largely invariant to inhibition protocol (Nakayama et al., 2018). Nonetheless, future studies could further examine the time course on which mPFC signals contribute to learning.

Our experiments indicate dopamine responses as the neuronal substrate for confidence-dependent learning. Consistent with previous reports, we found that VTA dopamine responses to outcomes reflect reward size and correlate with future choices (Bayer and Glimcher, 2005, Schultz, 2015). Moreover, we found that these responses also reflect confidence in achieving the reward and that confidence-dependent dopamine responses correlate with future choices. Our optogenetic manipulation of dopamine responses at the time of outcome shows that these responses causally affect behavior: activation and suppression of dopamine activity shifted the psychometric curves in opposite directions.

These dopamine signals appear to be pushing the mice toward a model-free strategy even though a model-based one would be more efficient. Our observation that choices depend on past decision difficulty reveals that mice adopted a model-free strategy (Figure 1; Figure S1). This strategy is suboptimal in our task but might be beneficial in a natural setting where stimuli are correlated in time (Yu and Cohen, 2008). It is driven by the post-outcome responses of dopamine neurons, which provide causal teaching signals for model-free confidence-dependent learning. We observed these results in mice that exhibited stable behavior over the course of weeks. It is possible, however, that longer training periods would result in model-based behavior.

Dopamine responses reflected sensory confidence and reward value also prior to outcome but did not play a causal role. The fact that pre-outcome activity is causal in mPFC but not in VTA suggests that the brain interprets mPFC activity as predicted value and VTA activity as prediction error. In our task, interfering before outcome with prediction error would not have much effect. Perhaps in a different task, where observing a stimulus is itself the result of a previous decision, the pre-outcome activity of dopamine neurons would cause learning.

The similarities and differences that we observed in mPFC neurons and VTA dopamine neurons suggest how they might be functionally related. Dopamine neurons receive predictive value signals from mPFC and subsequently compute prediction errors. Indeed, learning was affected by manipulation of predicted value signaled by mPFC neurons but not dopamine neurons. A causal role of frontal cortex in shaping dopamine responses would be consistent with anatomical projections (Beier et al., 2015, Carr and Sesack, 2000, Morales and Margolis, 2017), with simultaneous frontal-VTA recordings (Fujisawa and Buzsáki, 2011), and with recordings following pharmacological manipulations (Starkweather et al., 2018). These frontal projections to VTA may, in particular, affect inhibitory neurons, which could then play a role in subtracting predicted value from observed reward (Eshel et al., 2015, Gao et al., 2007).

These advances notwithstanding, our work leaves some key long-standing questions unanswered. First, where are the learned values stored? Previous studies suggest striatal neurons and neurons in frontal cortex are plausible candidates, because they encode the learned value of stimuli and actions and they receive strong dopamine projections (Ding and Gold, 2013, Lee et al., 2012, Padoa-Schioppa and Assad, 2006, Samejima et al., 2005, Sul et al., 2010, Tai et al., 2012). Second, where are the choices made, and how do they inform predicted value signals in mPFC? In a purely visual version of our task, the decisions are encoded in the activity of very sparse neurons in a distributed network across frontal cortex, motor cortex, striatum, and midbrain regions (Steinmetz et al., 2018). We speculate that a similar network may serve the choices in our task and provide signals that feed into mPFC for computing the predicted value of choice.

Neuroscience has at first studied decisions informed by perception and rewards in separate behavioral tasks, yielding data and models that are elegant yet separated (Sugrue et al., 2005, Summerfield and Tsetsos, 2012). Our work combines these approaches and offers a framework for understanding decisions guided by reward value and sensory evidence. This framework reveals how the brain uses both sensory confidence and reward value to drive learning, so that learning is strongest when rewards are obtained by making a hard decision.

## STAR★Methods

### Key Resources Table

**Table:** 

REAGENT or RESOURCE	SOURCE	IDENTIFIER
**Antibodies**		

Primary Anti-TH	Newmarket Scientific	22941
Secondary Alexa Goat anti-mouse conjugate (Alexa Fluor 594)	Life-Tech	A-11032
Anti-GFP antibody	Abcam	ab6556
Alexa Goat Anti RABBIT (Alexa Fluor 488)	Life-Tech	A-11034
Alexa Goat anti-mouse conjugate (Alexa Fluor 594)	Life-Tech	A-11032

**Virus Strains**		

AAV1.Syn.Flex.GCaMP6m.WPRE.SV40	Penn Vector Core	N/A
AAV5.EF1a.DIO.hChr2(H134R)-eYFP.WPRE	Gift from Karl Deisseroth (Addgene viral prep # 20298_AAV5); http://addgene.org/20298; RRID:Addgene_20298)	20298_AAV5
rAAV5/EF1a-DIO-eArch3.0-eYFP	University of North Carolina Vector Core	N/A

**Mice**		

C57/BL6J	N/A	N/A
B6.SJLSlc6a3tm1.1(cre)Bkmn/J	Jax	6660
B6.129P2-Pvalb^tm1(cre)Arbr^/J	Jax	8069

**Software and Algorithms**		

MATLAB	Mathworks	2016
ImageJ	NIH	https://imagej.nih.gov/ij
Signals	https://github.com/dendritic/signals	N/A

### Lead Contact and Materials Availability

Further information and requests should be directed to and will be fulfilled by the Lead Contact, Armin Lak (armin.lak@dpag.ox.ac.uk). This study did not generate new unique reagents.

### Experimental Model and Subject Details

The data presented here was collected from 33 mice (19 male) aged between 10-24 weeks. Wild-type C57/BL6J mice, DAT-Cre mice backcrossed with C57/BL6J mice (B6.SJLSlc6a3tm1.1(cre)Bkmn/J) and Pvalb-Cre mice backcrossed with C57/BL6J (B6.129P2-Pvalb^tm1(cre)Arbr^/J) were used. All experiments were conducted according to the UK Animals Scientific Procedures Act (1986) under appropriate project and personal licenses.

### Method Details

#### Surgeries

All mice were first implanted with a custom metal head plate. To do so, the animals were anesthetized with isoflurane, and were kept on a feedback-controlled heating pad (ATC2000, World Precision Instruments, Inc.). Hair overlying the skull was shaved and the skin and the muscles over the central part of the skull were removed. The skull was thoroughly washed with saline, followed by cleaning with sterile cortex buffer. The head plate was attached to the bone posterior to bregma using dental cement (Super-Bond C&B; Sun Medical). For electrophysiological experiments, we covered the exposed bone with Kwik-Cast (World Precision Instruments, Inc.), trained the animals in the behavioral task in the following weeks, and subsequently performed a craniotomy over the frontal cortex for lowering the silicon probes. For fiber photometry and optogenetic experiments, after the head plate fixation, we made a craniotomy over the target area (mPFC or VTA) and injected viral constructs followed by implantation of the optical fiber, which was secured to the head plate and skull using dental cement. Post-operative pain was prevented with Rimadyl on the three following days.

#### Behavioral tasks

Behavioral training started at least 7 days after the head plate implantation surgery. For mice which received viral injection, training started 2 weeks after the surgery. Animals were handled and acclimatized to head fixation for 3 days, and were then trained in a 2-alternative forced choice visual detection task (Burgess et al., 2017). After the mouse kept the wheel still for at least 0.5 s, a sinusoidal grating stimulus of varying contrast appeared on either the left or right monitor, together with a brief tone (0.1 s, 12 kHz) indicating that the trial had started. The mouse could immediately report its decision by turning the wheel located underneath its forepaws. Wheel movements drove the stimulus on the monitor, and a reward was delivered if the stimulus reached the center of the middle monitor (a correct trial), but a 2 s white noise was played if the stimulus reached the center of the either left or right monitors (an error trial). The inter trial interval was set to 3 s. As previously reported, well-trained mice often reported their decisions using fast stereotypical wheel movements (Burgess et al., 2017). In the initial days of the training (first 4 to 7 days), stimuli had contrast = 1. Lower-contrast stimuli were introduced when the animal reached the performance of ∼70%. After 2-3 weeks of training, the task typically included 7 levels of contrast (3 on the left, 3 on the right and zero contrast) which were presented in a random order across trials with equal probability. We finally introduced unequal water rewards for correct decisions: in consecutive blocks of 50-350 trials (drawn from a uniform distribution), correct decisions to one side (left or right) were rewarded with larger reward (2.4 μL versus 1.2 μL of water) (Figure 1).

Experiments involving optogenetic manipulation of mPFC neurons or VTA dopamine neurons had the same timeline as described above (Figures 4 and 5). In experiments involving fiber photometry, the task timeline slightly differed from above, allowing longer temporal separation of stimulus, action and outcome (Figure 3). In these experiments, wheel movements immediately after the visual stimulus did not move the stimulus on the monitor and did not result in a decision (open-loop condition). Instead, an auditory go cue (0.1 s) which was played 0.6-1.8 s after the stimulus onset started the closed-loop during which animals could report the decision. Wheel movements prior to go cue did not terminate the trial and we did not exclude these trials from our analysis (excluding these trials did not affect our results). In these experiments, we defined the action time as the onset of first wheel movement after the stimulus onset. In all experiments, reaction times were measured from the onset of visual stimulus till the onset of the first wheel movement.

The behavioral experiments were controlled by custom-made software written in MATLAB (Mathworks) which is freely available (Bhagat et al., 2019). Instructions for hardware assembly are also freely available (https://www.ucl.ac.uk/cortexlab/tools/wheel).

#### Electrophysiological experiments

We recorded neuronal activity in prelimbic region of mPFC using multi-shank silicon probes in wild-type C57/BL6J mice. We implanted the animals after they fully learned to perform the task, performing the final stage of the behavioral task (including block switches) with performance above 70% for at least three sessions. A 32-channel, 2 shank silicon probe (Cambridge NeuroTech) was mounted on a moveable miniature Microdrive (Cambridge NeuroTech) and implanted it into mPFC (n = 6 mice). On the implantation day, we removed the Kwik-Cast cover from the skull and drilled a small incision in the cranium over the frontal cortex, ML = 0.3 mm, AP = 1.8 mm (burr #19007–07, Fine Science Tools). The brain was protected with Ringer solution. We lowered the probe through the intact dura using a manipulator (PatchStar, Scientifica) to 1.4 mm from the dura surface. The final approach toward the target depth (the last 100–200 μm) was performed at a low speed (2–4 μm/sec), to minimize potential damage to brain tissue. Once the probe was in its required position, we waited 10 minutes to let the brain recover from the insertion and fixed the Microdrive on the head plate using dental cement. For reference signal we used a skull screw implanted on the skull ∼3-4 mm posterior to the recording site. At the end of each recording day we lowered the Microdrive 100 μm.

Recordings were performed using OpenEphys system. Broadband activity was sampled at 30 kHz (band pass filtered between 1 Hz and 7.5 kHz by the amplifier) and stored for offline analysis. Recorded spikes were sorted with KlustaSuite (Rossant et al., 2016). Manual spike sorting was performed oblivious to task-related responses of the units.

#### Fiber photometry experiments

To measure the activity of dopamine neurons, we employed fiber photometry (Gunaydin et al., 2014, Lerner et al., 2015). We injected 0.5 μL of diluted viral construct (AAV1.Syn.Flex.GCaMP6m.WPRE.SV40) into the VTA:SNc (ML:0.5 mm from midline, AP: −3 mm from bregma and DV:-4.4 mm from the dura) of DAT-Cre mice backcrossed with C57/BL6J mice (B6.SJLSlc6a3tm1.1(cre)Bkmn/J). We implanted an optical fiber (400 μm, Doric Lenses Inc.) over the VTA, with the tip 0.05 mm above the injection site. We used a single chronically implanted optical fiber to deliver excitation light and collect emitted fluorescence. We used multiple excitation wavelengths (465 and 405 nm) modulated at distinct carrier frequencies (214 and 530 Hz) to allow for ratiometric measurements. Light collection, filtering, and demodulation were performed as previously described (Lerner et al., 2015) using Doric photometry setup and Doric Neuroscience Studio Software (Doric Lenses Inc.). For each behavioral session, least-squares linear fit was applied to the 405nm control signal, and the ΔF/F time series was then calculated as ((490nm signal – fitted 405nm signal) / fitted 405nm signal). All analyses were done by calculating z-scored ΔF/F.

#### Optogenetic experiments

##### Optogenetic manipulation of mPFC neurons

For suppressing mPFC responses, we injected 0.5 μL of diluted viral construct containing ChR2 (AAV5.EF1a.DIO.hChr2(H134R)-eYFP.WPRE) unilaterally into the mPFC (ML:0.3 mm, AP: 1.8 mm from bregma and DV:-1.6 mm from the dura) of Pvalb-Cre mice backcrossed with C57/BL6J (B6.129P2-Pvalb^tm1(cre)Arbr^/J). We implanted an optical fiber (200 μm, Doric Lenses Inc.) over the mPFC, with its tip staying 0.4 mm above the injection site. We waited 2 weeks for virus expression and then started the behavioral training. After achieving stable task performance using symmetric water rewards, we introduced laser pulses which had following parameters: 473 nm (Laserglow LTD), number of pulses: 12, each pulse lasting 10 ms and separated by 30 ms, laser power: ∼2-3 mW (measured at the fiber tip). The laser pulses were applied either from the stimulus onset (Figure 4; Figure S4) or during the outcome (Figure S4). Manipulation at the time of the stimulus included three types of experiments: a) in 40% of randomly chosen trials in the task that had blocks of 50-350 trials with unequal rewards, b) in the task that had blocks of 50-350 trials with unequal rewards each of them with or without laser pulse at the stimulus time, making four types of blocks, c) in 40% of randomly chosen trials of a purely visual task (with symmetric and stable rewards). In the experiments involving manipulations at the trial outcome, in consecutive blocks of 50-350 trials, correct decisions to one side, L or R, were paired with laser pulses (Figure S4).

##### Optogenetic manipulation of VTA dopamine neurons

For activating or suppressing dopamine neurons, We injected 0.5 μL of diluted viral constructs containing ChR2 (AAV5.EF1a.DIO.hChr2(H134R)-eYFP.WPRE) or Arch3 (rAAV5/EF1a-DIO-eArch3.0-eYFP) unilaterally into VTA:SNc (ML:0.5 mm from midline, AP: −3 mm from bregma and DV:-4.4 mm from the dura) of DAT-Cre mice backcrossed with C57/BL6J mice (B6.SJLSlc6a3tm1.1(cre)Bkmn/J). We implanted an optical fiber (200 μm, Doric Lenses Inc.) over the VTA, with its tip staying 0.4 mm above the injection site. We waited 2 weeks for virus expression and then started the behavioral training. After achieving stable task performance using symmetric water rewards, we introduced laser pulses which had the following parameters: 473 nm and 532 nm for ChR2 and Arch3, respectively (Laserglow LTD), number of pulses: 12, each pulse lasting 10 ms and separated by 30 ms, laser power: ∼8 mW (measured at the fiber tip). For the suppression experiment using Arch3, in few sessions we used a single 300 ms long pulse. The laser pulses were applied either 0.4 s prior to the stimulus (Figure S5), exactly at the time of the stimulus (Figure 5; Figure S5), or at the time of the reward (Figure 5; Figure S5). For experiments involving activation of dopamine neurons prior to the stimulus onset, in 40% of randomly chosen trials, we delivered laser pulses. For experiments involving activation of dopamine neurons at the stimulus onset, we either applied pulses in 40% of randomly chosen trials (Figure S5) or in blocks of 50-350 trials (Figure 5). In the experiments involving manipulation of dopamine activity at the trial outcome, in consecutive blocks of 50-350 trials, correct decisions to one side, L or R, were paired with laser pulses (Figure 5). In experiments involving trial-by-trial manipulations at the trial outcome (rather than blocks of trials), in 30% of randomly chosen correct trials, the reward was paired with laser pulses (Figure S5). In both these experiments the laser was turned on simultaneously with the TTL signal that opened the water valve.

#### Histology and anatomical verifications

To verify expression of viral constructs we performed histological examination. Animals were deeply anesthetized and perfused, brains were post-fixed, and 60 μm coronal sections were collected. For optogenetic experiments on mPFC, we immunostained with antibody to eYFP and secondary antibodies labeled with Alexa Fluor 488 (Figure 4). For experiments on dopamine neurons (both photometry and optogenetic), sections were immunostained with antibody to TH and secondary antibodies labeled with Alexa Fluor 594. For animals injected with ChR2 or Arch3 constructs into the VTA, we also immunostained with an antibody to eYFP and secondary antibodies labeled with Alexa Fluor 488 (Figure 5; Figure S5). We confirmed viral expression in all animals with ChR2 injections into the mPFC and in 14 (out of 15) mice injected with ChR2, Arch3 or GCaMP6M.

The anatomical location of implanted optical fibers was determined from the tip of the longest fiber track found, and matched with the corresponding Paxinos atlas slide (Figures 3, 4, and 5; Figures S4 and S5). To determine the position of silicon probes in mPFC, coronal sections were stained for GFAP and matched to the corresponding Paxinos atlas (Figure 2A). Confocal images from the sections were obtained using Zeiss 880 Airyscan microscope.

### Quantification and Statistical Analysis

#### Behavioral modeling

To estimate the hidden variables that could underlie learning and decisions in our tasks, we adopted a reinforcement learning model which we developed previously (Lak et al., 2017). In our task, knowing the state of the trial (L or R) is only partially observable, and it depends on the stimulus contrast.

In keeping with the standard psychophysical treatments of sensory noise, the model assumes that the internal estimate of the stimulus, $\hat{s}$, is normally distributed with constant variance around the true stimulus contrast: $p\left(\hat{s}|s\right)=N\left(\hat{s};s,\sigma^{2}\right)$. In the Bayesian view, the observer’s belief about the stimulus s is not limited to a single estimated value $\hat{s}$. Instead, $\hat{s}$ parameterizes a belief distribution over all possible values of s that are consistent with the sensory evidence. The optimal form for this belief distribution is given by Bayes rule:

$$p\left(s|\hat{s}\right)=\frac{p\left(\hat{s}|s\right).p\left(s\right)}{p\left(\hat{s}\right)}$$

We assume that the prior belief about s is uniform, which implies that this optimal belief will also be Gaussian, with the same variance as the sensory noise distribution, and mean given by $\hat{s}$: $p\left(s|\hat{s}\right)=N\left(s;\hat{s},\sigma^{2}\right)$. From this, the agent computes a belief, i.e., the probability that the stimulus was indeed on the right side of the monitor, $p_{R}=p\left(s>0|\hat{s}\right)$, according to:

$$p_{R}=\int_{0}^{\infty }p\left(s|\hat{s}\right)\;ds$$

$p_{R}$ represents the trial-by-trial probability of the stimulus being on the right side (and $p_{L}=1-p_{R}$ represents the probability of it being on the left).

The expected values of the two choices L and R are computed as $Q_{L}=p_{L}V_{L}$ and $Q_{R}=p_{R}V_{R}$, where $V_{L}$ and $V_{R}$ represent the stored values of L and R actions. To choose between the two options, we used an argmax rule which selects the action with higher expected value deterministically (Figure 1). Using other decision functions such as softmax did not substantially change our results. The outcome of this is thus the choice (L or R), its associated confidence $p_{C}$, and its predicted value $Q_{C}$.

$$Q_{C}=\{\begin{array}{c} Q_{L}\;if\;choice=L\\ Q_{R}\;if\;choice=R \end{array}$$

When the trial begins, i.e., when the auditory cue indicates that the trial has started, the expected reward prior to any information about the stimulus is $V_{onset\;tone}=\left(V_{L}+V_{R}\right)/2$. Upon observing the stimulus and making a choice, the prediction error signal is: $Q_{C}-V_{onset\;tone}$. After receiving the reward, r, the reward prediction error is $\delta =r-Q_{C}$.

Given this prediction error the value of the chosen action will be updated according to:

$$V_{C}\leftarrow V_{C}+\alpha .\delta$$

where α is the learning rate. For simplicity, the model does not include temporal discounting.

The model’s estimates of both $Q_{C}$ and δ, depend on stimulus contrast, reward size, and whether the choice is correct (Figure 1; Figure S1). $Q_{C}$ grows with the stimulus contrast as well as the size of reward. Perhaps less intuitively, however, the dependence of $Q_{C}$ on contrast is reversed on error trials (Figure 1J, *red curve*). This effect is easily understood if $V_{L}=V_{R}$. In this case, errors are entirely due to wrong sensory estimates of $p_{L}$ and $p_{R}$. If a stimulus is on the R, the observer chooses L only if $p_{c}=p_{L}>p_{R}$. In high-contrast trials, this occurs rarely and by a small margin (Kepecs and Mainen, 2012, Lak et al., 2017), so $p_{c}\approx 0.5$ and $Q_{C}$ is low. At lower contrast, instead, this can occur more often and with $p_{c}\gg 0.5$, so $Q_{C}$ is higher.

##### Model fitting

The experiments included sessions with blocks of trials with unequal water rewards and sessions with no reward size manipulation. In the optogenetic experiments, these sessions could include suppression of mPFC neurons or activation/suppression of VTA dopamine neurons.

We fitted our model as well as reduced model variants on choices acquired in the task with unequal water rewards and cross-validated the necessity of model parameters. We then used the model that could best account for the data and fitted it on the experiments that included optogenetic manipulations.

##### Experiments with unequal water reward

For fitting, we set the value of smaller water reward to 1. Thus, the payoff matrix for blocks with larger reward on the left or right, respectively, are:

$$outcome:\left[\begin{array}{cc} 0 & 1\\ 1+x & 0 \end{array}\right],outcome:\left[\begin{array}{cc} 0 & 1+x\\ 1 & 0 \end{array}\right]$$

where x, a constant, represents the value of extra drop of water. We set the payoff for incorrect decisions to zero in all our model fitting.

We fitted the model as well as reduced model variants on the decisions of mice in the task with unequal water rewards, and cross-validated the necessity of model parameters (Figure S1). As described above, the full model included the following parameters: $\sigma^{2}$, x, α. Each reduced model did not include one of these parameters. For $\sigma^{2}$, one reduced model was set to have $\sigma^{2}=0$, representing a model with no sensory noise, and the other reduced model was set to have $\sigma^{2}=\infty$, representing a model with extremely large sensory noise. For cross-validated fitting, we divided sessions of each mouse to 3 and performed a 3-fold cross validation. We performed the fit and parameter estimation on the training sessions and used the estimated parameters against the test sessions for computing goodness of fit. For fitting, we performed exhaustive search in the parameter space expanding large value range for each of the parameters to find the best set of model parameters that account for the observed decisions. We searched the following parameter space: α=0:0.05:0.95, $\sigma^{2}=0.04:0.04:0.8$ and x=−5:0.2:10. To do so, for each possible combination of these parameters, we repeatedly fed the sequences of stimuli that each mouse experienced to the model, observed decisions (iteration = 1000), and averaged across the iterations to compute the probability that model made a leftward and rightward decision $\left(\hat{P}\left(L\right),\hat{P}\left(R\right)\right)$ for each trial. We then calculated the negative log likelihood (NLL) as the average of $–\log\left(\hat{P}\left(\text{choice}\right)\right)$, where choice indicates the mouse’s decision in each trial (Figure S1). The set of parameters that gave the lowest NLL were used to compute goodness of fit in the test sessions (3-fold cross-validation).

##### Manipulation of mPFC activity

For experiments including suppression of the mPFC at the stimulus onset, we allowed the model to add a constant to the predicted value of the choice $Q_{C}$. A negative constant resulted in lower predicted value and hence increased prediction error after receiving a reward (Figure S4). We fitted the model on choices as described above. The other possible way in which the model could be modified to show a larger shift in the psychometric curve is by simulating the effect of mPFC suppression as increasing the sensory noise $\left(\sigma^{2}\right)$. However, this also results in curves with shallower slopes which we did not observe in the data (Figure 4; Figure S4).

##### Manipulation of dopamine activity

For experiments including suppression or activation of dopamine neurons at the outcome time, we allowed the model to add a constant to the reward prediction error δ. This constant was negative for the experiment with dopamine suppression, and was positive for the experiment with dopamine activation (Figure S5). We fitted the model on choices as described above.

##### Optimal observer model fitting

We constructed an alternative class of model that optimally performs our task. This observer leverages the structure of the task, i.e., it knows that only two reward sizes are available and that they switch side occasionally. The observer would thus only need to infer whether it is in the left or the right block, given the sequence of outcomes in the previous trials. To do so we used a hidden Markov model (MATLAB HMM toolbox). The model estimates the trial-by-trial probability that the current state is left or right block $p_{\left(S=L\right)}$ and $p_{\left(S=R\right)}$, respectively, given a state transition matrix and an observation matrix. The state transition matrix defines the probability of block switch, which can be calculated from the number of block switches and number of trials in each dataset. The observation matrix defines the probability of observed outcomes (no reward, small reward and larger reward) given each state. The model computes the expected value of left and right actions according to:

$Q_{L}=p_{L}\left(p_{\left(S=L\right)}r_{\left(a=L,s=L\right)}+p_{\left(S=R\right)}r_{\left(a=L,s=R\right)}\right)$ and $Q_{R}=p_{R}\left(p_{\left(S=L\right)}r_{\left(a=R,s=L\right)}+p_{\left(S=R\right)}r_{\left(a=R,s=R\right)}\right)$, where $p_{L}$and $p_{R}$ are estimated as described in the reinforcement learning model section, $r_{\left(a=L,s=L\right)}$ indicates the size of reward available for left action in the L block and $p_{\left(S=L\right)}$ and $p_{\left(S=R\right)}$ are the probabilities that the current trial belongs to L or R block, estimated using the hidden Markov model. This model learns about the blocks from any rewarded trials (both small and large rewards). This learning is, however, not influenced by the sensory confidence. When emission matrix is set optimally (i.e., in the left block the probability of large reward on the right is zero, p(r=large|a=R,s=L)=0, etc.), the model learns the block switch after only one rewarded trial. However, we observed that mice took several trials to learn the block switch (Figure S1). Thus, for the fitting purpose, we considered that the observation matrix is noisy (p(r=large|a=R,s=L)=β). An intuition behind this could be that the mouse does not always accurately detect the size of reward, and is hence slightly confused about the size of rewards which are available for L and R choices in each block; β determines this noise level. We estimated β for each animal using exhaustive search, as described in the previous section. After fitting, the model could account for the dependence of decisions on past rewards and current sensation (Figure S1), but not for the dependence of choices on decision confidence in the previous trial (Figure S1).

#### Additional behavioral analyses

##### The effect of sensory confidence on learning

To isolate the effect of sensory confidence on learning (Figures 1E and 1F; Figures S1E and S1F), we computed ‘Rightward (%)’ for each level of stimulus contrast conditional on preceding trial being a rewarded trial with either difficult or easy stimuli on the left or the right, resulting in four curves (Figures 1E and 1F). We then computed the difference between the two post-difficult curves and the difference between the two post-easy curves to compute Δ Rightward (%), as shown in Figure 1E and Figure S1E. This analysis involved an intermediate correction which ensures that the effect of past stimulus difficulty on choices is not due to slowly fluctuating bias over trials (i.e., serial correlation of choices due to slow side bias). This normalization procedure estimated the degree of choice bias in relation to possible bias in previous trials. We reasoned that slow fluctuations are, by definition, slower than one trial, and hence should be largely similar in adjacent trials. This assumption leads to a simple strategy to correct for possible drifts and isolate psychometric curve shifts due to past sensory confidence. To do so, we estimated ‘Δ Rightward (%)’ conditional on preceding trial being a rewarded trial with either easy or difficult stimulus (as described above), and we also estimated ‘Δ Rightward (%)’ conditional on the following trial being a rewarded one again with either easy or difficult stimulus. We then subtracted the latter from the former (Figure 1E; Figure S1E). This removes the effect of slow response bias and provides an estimate of how the current trial influences choices in the next trial.

##### Fitting of conventional psychometric function

In order to test the effect of optogenetic manipulation on decisions, in addition to the model fitting described above, we used conventional psychometric fitting, Palamedes toolbox (Prins and Kingdom, 2018), and tested whether the optogenetic manipulations influenced the slope and bias parameters of these fits. None of the manipulations influenced psychometric slopes, and the effect of manipulations on the bias was fully consistent with the results from our reinforcement model fittings.

For analysis of reaction times, the reaction times from each session were first z-scored before averaging across sessions and animals.

#### Neuronal regression analysis

In order to quantify how each task event (stimulus, action, outcome) contributes to neuronal activity, and, the extent to which trial-by-trial variation in neuronal responses reflects animal’s estimate of pending reward and prediction error, we set up a neuronal response model (Park et al., 2014) (Figures 2, 3, S2, and S3).

We modeled the spiking activity of a neuron during trial j, which we denote $R_{j}\left(t\right)$ as

$$R_{j}\left(t\right)=S_{j}K_{s}\left(t\right)*X_{j}^{s}\left(t\right)+A_{j}K_{a}\left(t\right)*X_{j}^{a}\left(t\right)+O_{j}K_{o}\left(t\right)*X_{j}^{o}\left(t\right)$$

In the above equation, $K_{s}\left(t\right)$, $K_{a}\left(t\right)$ and $K_{o}\left(t\right)$ are the profiles (kernels) representing the response to the visual stimulus, the action, and the outcome. $X_{j}^{s}\left(t\right)$, $X_{j}^{a}\left(t\right)$ and $X_{j}^{o}\left(t\right)$ are indicator functions which signify the time point at which the stimulus, action and outcome occurred during trial j. $S_{j}$, $A_{j}$ and $O_{j}$ are multiplicative coefficients which scale the corresponding profile on each trial and ^∗^ represents convolution. Therefore, the model represents neuronal responses as the sum of the convolution of each task event with a profile corresponding to that event, which its size was scaled in each trial with a coefficient to optimally fit the observed response. Given the temporal variability of task events in different trials, the profile for a particular task event reflects isolated average neuronal response to that event with minimal influence from nearby events. The coefficients provide trial-by-trial estimates of neuronal activity for each neuron.

The model was fit and cross-validated using an iterative procedure, where each iteration consisted of two steps. In the first step the coefficients $S_{j}$, $A_{j}$ and $O_{j}$ were kept fixed and the profile shapes were fitted using linear regression. Profiles were fitted on 80% of trials and were then tested against the remaining 20% test trials (5-fold cross-validation). In the second step, the profiles were fixed and the coefficients that optimized the fit to experimental data were calculated, also using linear regression. Five iterations were performed. In the first iteration, the coefficients were initialized with value of 1. We applied the same analysis on the GCaMP responses (Figure 3; Figure S3).

We defined the duration of each profile to capture the neuronal responses prior to or after that event and selected longer profile durations for the GCaMP data to account for Ca^+2^ transients (mPFC spike data: stimulus profile: 0 to 0.6 s, action profile: −0.4 to 0.2 s, outcome profile: 0 to 0.6 s; GCaMP data: stimulus profile: 0 to 2 s, action profile: −1 to 0.2 s, outcome profile: 0 to 3 s, where in all cases 0 was the onset of the event). For both spiking and GCaMP data, the neuronal responses were averaged using a temporal window of 20 and 50 ms, respectively, and were then z-scored.

### Data and Code Availability

The datasets supporting the current study have not been deposited in a public repository because of large file size, but are available from the corresponding author on request.

## Supplemental Information

Document S1. Figures S1–S5

Document S2. Article plus Supplemental Information
