# Supplementary material for: Dopaminergic and Prefrontal Basis of Learning from Sensory Confidence and Reward Value
Source: Neuron. 2020 Feb 19;105(4):700–711.e6. doi: 10.1016/j.neuron.2019.11.018 (PMC7031700; doi:10.1016/j.neuron.2019.11.018)
Supplement: Document S1. Figures S1–S5 [file mmc1.pdf]

Neuron, Volume *105*

Supplemental Information

Dopaminergic and Prefrontal Basis of Learning

from Sensory Confidence and Reward Value

Armin Lak, Michael Okun, Morgane M. Moss, Harsha Gurnani, Karolina Farrell, Miles J. Wells, Charu Bai Reddy, Adam Kepecs, Kenneth D. Harris, and Matteo Carandini

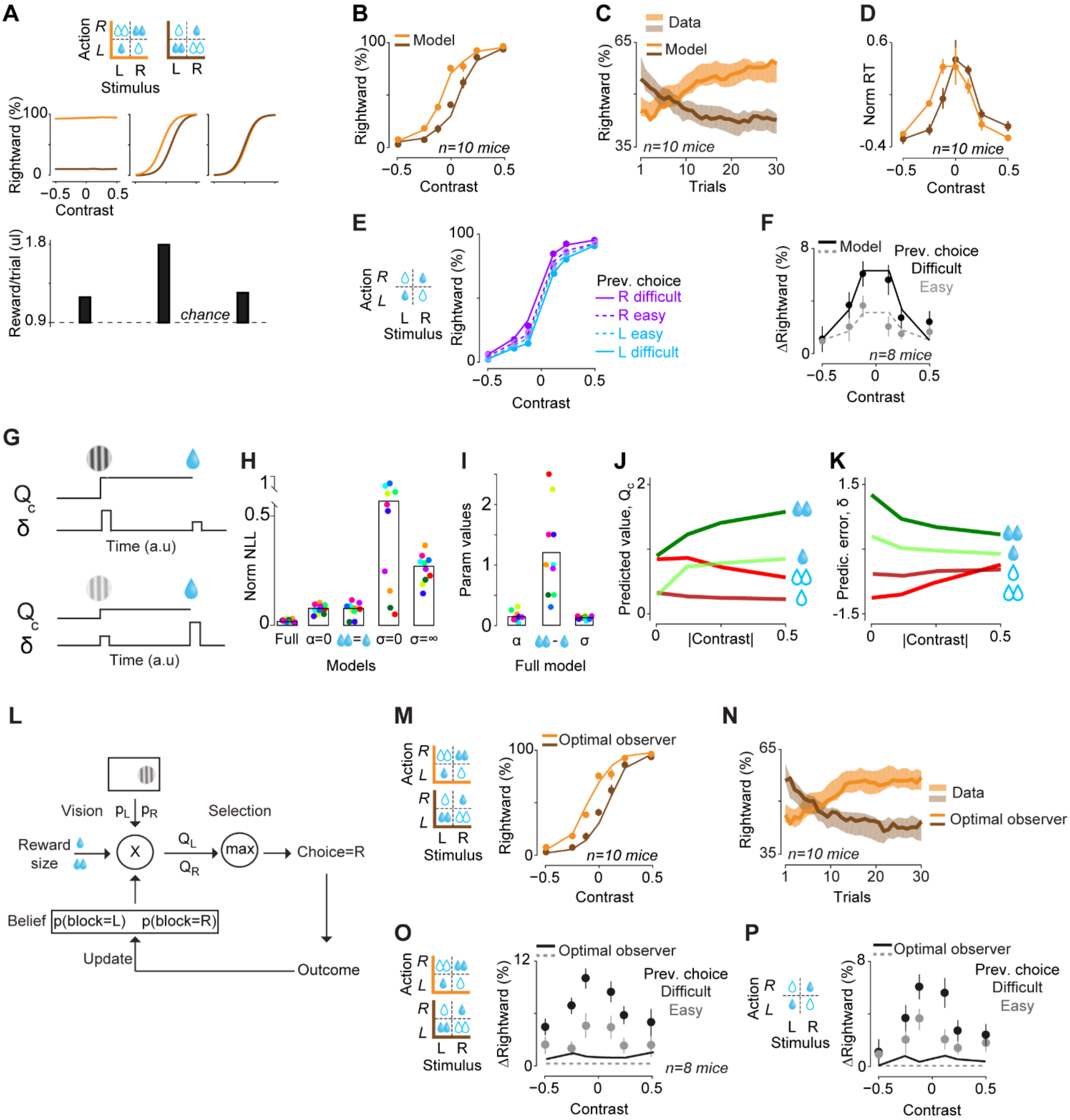

Figure S1. Related to Figure 1. Behavioral and computational signatures of decisions guided by reward values and sensory evidence.

(A) Simulation of three agents that performed the behavioral task (top row) and their average reward harvest during 5000 trials. Left: an agent that made frequent decisions towards the side paired with larger reward, regardless of the stimulus contrast. Middle: an agent that integrated past rewards and sensory evidence. Right: an agent that made decisions only according to the sensory stimulus, ignoring the reward size. Chance level in the lower panel indicates the reward harvest of an agent that made left and right decisions in a random fashion. Simulations are performed using the model described in Figure 1. For the simulation on the left, σ^2=2 and α=0.2 so the model could not perform the visual task but could learn from the past rewards. For the simulation in the middle panel we set σ^2=0.2 and α=0.2. For the simulation on the right, we set σ^2=0.2 and α=0, so the model could perform visual detection but could not learn from past rewards.

(B) Average performance of mice in blocks with large reward on the left (brown) or on the right (orange). Curves are model fits on the data.

(C) Average learning curves following block switch for mice (shaded regions, mean $\pm$ s.e.) and model predictions (curves). Mice were gradual in their learning of reward value. For instance, they took ~12 trials to shift their low-contrast decisions by 10% towards the side newly paired with the larger reward.

(D) Reaction time of animals. Reaction times were z-scored before averaging across sessions and mice. Shorter reaction times were seen for high stimulus contrast and for stimuli on the side indicating larger reward size for the current block ($P < 10^{-10}$, 2-way ANOVA). Error bars: s.e. across mice.

(E) Performance of an example animal as a function of the difficulty of previous correct choices in the visual decision task with no reward manipulation.

(F) Similar to (Figure 1F) but for the experiment without reward manipulation.

(G) Schematic of predicted value of choice, $Q_{C,}$ and prediction error, δ, of the model in trials where a high contrast or low contrast stimulus led to the same reward.

(H) Cross-validated negative log likelihood of different variants of the model. Full model contained all parameters, while each reduced model excluded one of them. Circles with different colors represent different animals.

(I) Estimated parameters of the best model, i.e. the full model.

(J) Averaged estimates of Q_C as a function of absolute contrast (i.e. regardless of side), for correct decisions towards the large-reward side (dark green) and correct decisions towards the small-reward side (light green), error trials towards the large-reward side (red) and error trials toward the small-reward side (dark red).

(K) Similar to (J) but for reward prediction error δ.

(L) Schematic of the optimal observer. The model uses three quantities to compute the expected value of left or right actions: the sensory evidence, the size of the rewards, and the probability (belief) that it is the left or right block. After making a choice, the model observes the outcome and updates the belief about which choice direction is associated with the larger reward. Receiving a small or large reward causes learning because they are informative about which side is paired with larger reward, but receiving no reward (error trial) is not informative (see Methods).

(M) The model accounts for the psychometric shifts in blocks with larger rewards in the left or right.

(N) The model accounts for learning curves after the block switch.

(O) The model does not account for the dependence of decisions on the difficulty of past sensory judgment. The curves are the model fits and the data are identical to those in (Figure 1F).

(P) Similar to (O) but for the task with no reward size manipulation. The data is identical to those in (F).

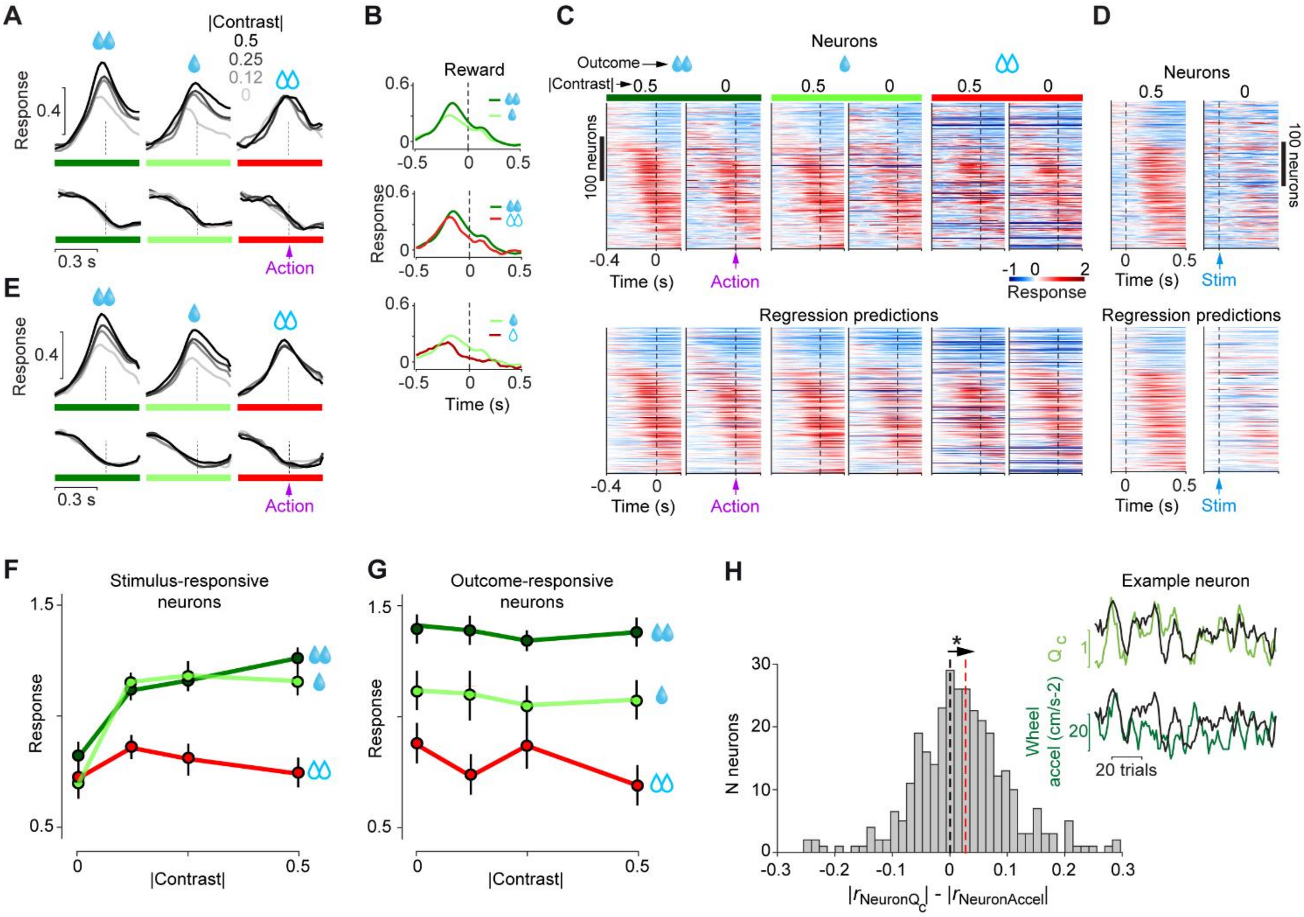

Figure S2. Related to Figure 2. mPFC neuronal responses during the task.

(A) Mean population activity triggered on action onset. Responses were z-scored before averaging. Only neurons with decreased activity at the time of action were included. Left: correct choices towards the large-reward side; Middle: correct choices towards the small-reward side; Right: incorrect choices towards the large-reward side. Shades of gray: stimulus contrast.

(B) Mean population activity triggered on outcome onset.

(C) Top: Normalized responses aligned to action onset. Different panels show all cells' responses averaged across trials of the contrast and reward value shown above the panels. Dark blue horizontal lines in error trial panels (rightmost) reflect the fact that in few recording sessions animal performed very few error trials. Bottom: Same as upper panels but for predictions of the regression that only included action events, i.e. only allowed action profiles and their coefficients.

(D) Top: normalized neuronal responses aligned to stimulus onset in all trials with |contrast| = 0.5 or 0. Bottom: these responses are accurately predicted by the regression that only included action events.

(E) Predictions of the regression triggered on action, as a function of stimulus contrast and trial type for neurons shown in (A).

(F) Average stimulus responses of neurons with significant stimulus profile as a function of stimulus contrast and reward size. Neurons which responded at the time of the stimulus encoded stimulus contrast and had lower responses in error trials than in correct trials but did not encode the size of the upcoming rewards (67/316 neurons stat), therefore did not reflect Q_C. G) Average outcome responses of neurons with significant outcome profile as a function of stimulus contrast and reward size. Neurons responding at the time of outcome encoded outcome value, i.e. large reward, small reward or no reward, independent of stimulus contrast (48/316 neurons, stat), and thus did not reflect δ.

(H) Correlation of mPFC neurons with trial-by-trial Q_C and trial-by-trial wheel acceleration during decision, as a proxy of response vigor. mPFC neurons were better correlated with Q_C compared to wheel acceleration (54 vs 22 neurons, P < 0.01, linear partial correlation). The inset shows the fluctuation of responses of an example neuron (black trace) vs estimated Q_C as well as wheel acceleration during decisions.

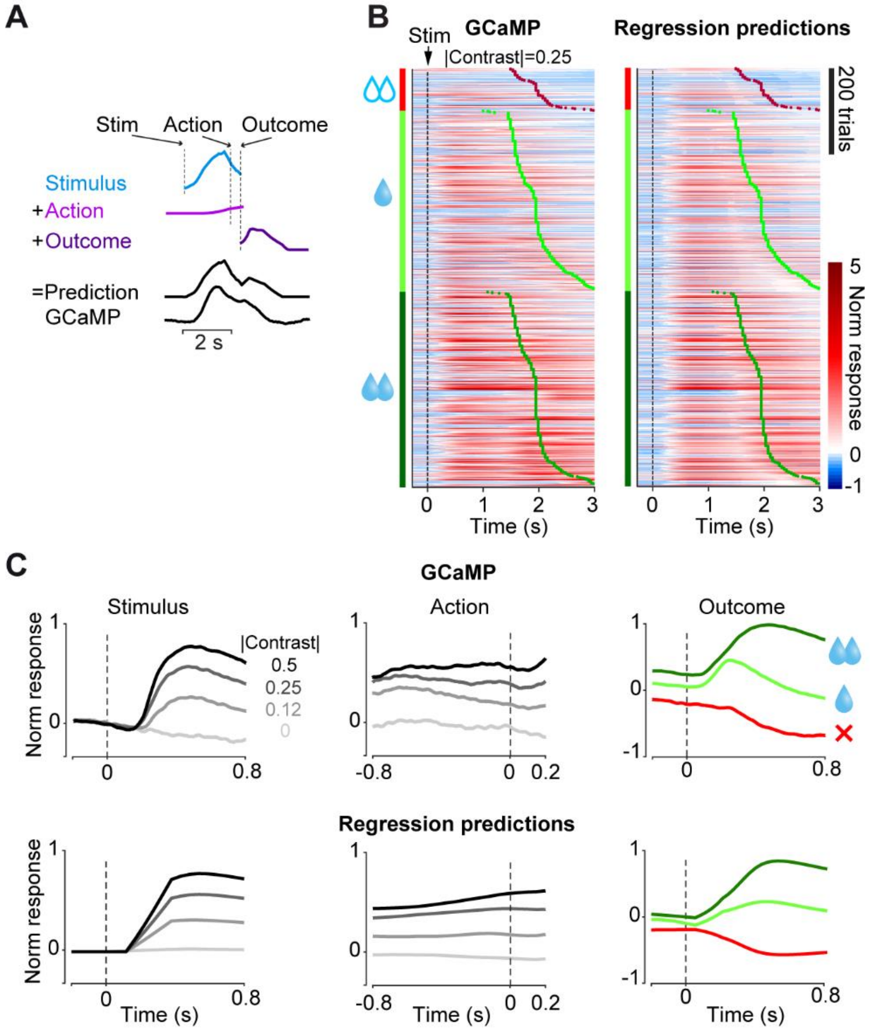

Figure S3. Related to Figure 3. Dopamine neuronal activity during the task.

(A) Schematic of regression analysis. The regression estimates a temporal profile for each task event, which are convolved with the event times, scaled in each trial with a coefficient and summed to produce regression predictions. The interval between the go cue and the action onset was, on average, 144 ms and thus in our regression we treated these as one event because slow time course of GCaMP might not dissociate them.

(B) Left: trial-by-trial dopamine responses (as in Figure 3C), aligned to the stimulus onset (dashed line), with trials arranged vertically by trial type and outcome time (red/green points). Right: predictions of the regression analysis that only included stimulus and outcome events.

(C) Mean dopamine activity aligned to the stimulus, action and outcome. Lower panels show the predictions of the regression analysis.

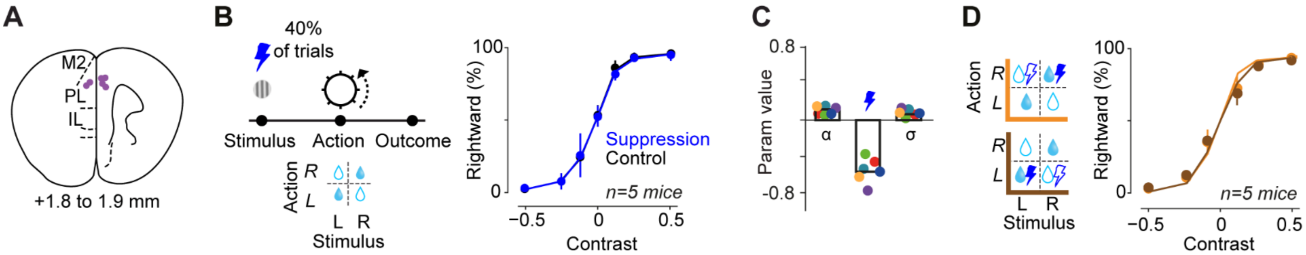

Figure S4. Related to Figure 4. Optogenetic manipulation of mPFC neurons during the task.

(A) Localization of implanted fiber tips from histological slices in each mouse.

(B) Optogenetic suppression of mPFC neurons at the onset of visual stimulus did not influence task performance (P = 0.97, 1-way ANOVA on the difference between datapoints, P = 0.87 signed rank test on the slope of psychometric curves) nor their associated reaction times (P = 0.84, 1-way ANOVA). Curves are model fits.

(C) Estimated model parameters for experiments in which mPFC was suppressed during the stimulus presentation in the task with unequal reward size. Optogenetic suppression reduced the estimates of predicted value of choice. Circles with different colors represent different animals.

(D) Optogenetic suppression of mPFC neurons during the outcome time did not influence task performance (P = 0.96, 1-way ANOVA) nor their associated reaction times (P = 0.4, 1-way ANOVA). The manipulations were performed in blocks of trials and the water rewards were equal across sides, as shown on the left column. See Figure 5D-F for similar experiments on VTA dopamine neurons.

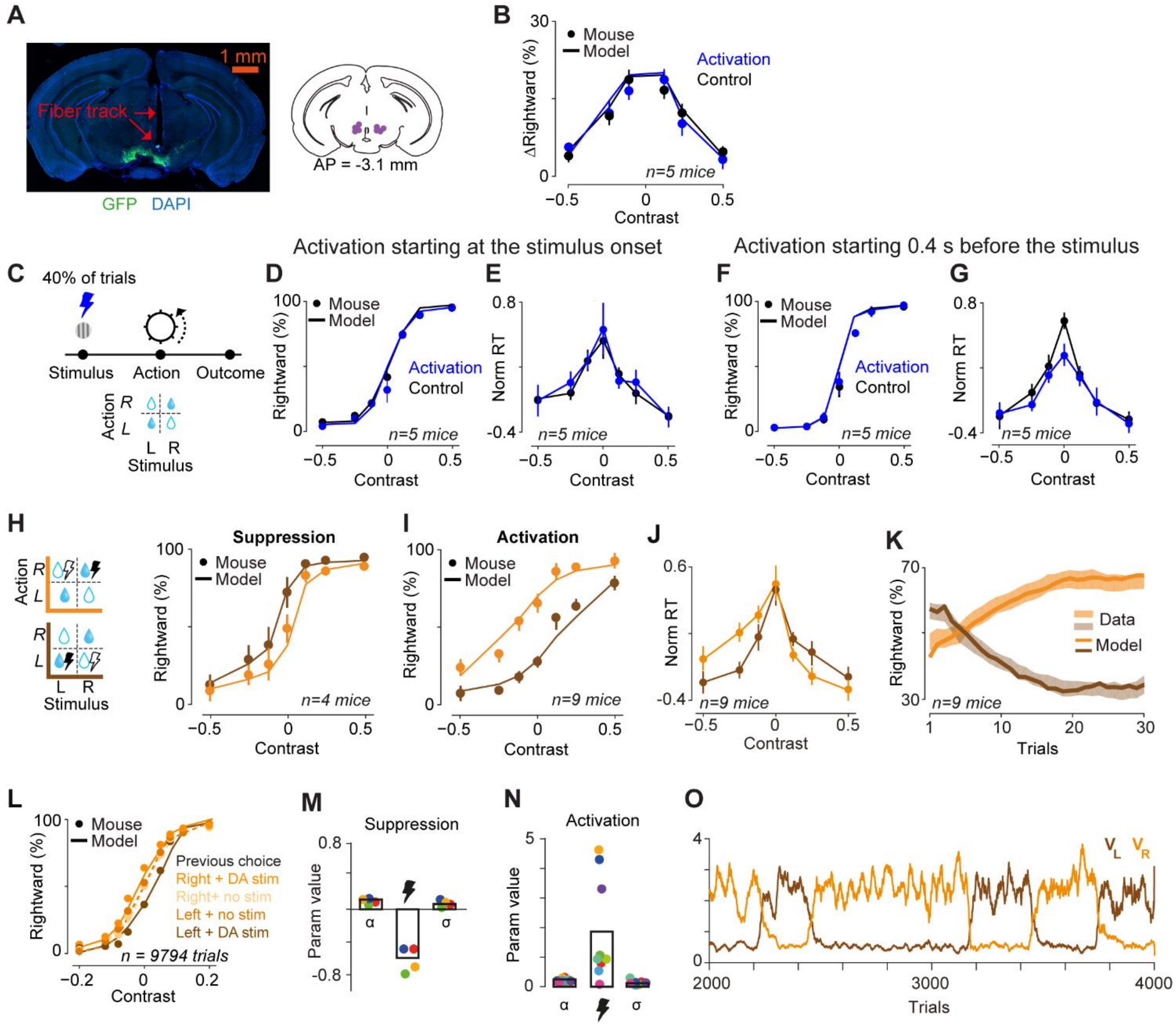

Figure S5. Related to Figure 5. Optogenetic manipulation of VTA dopamine activity during the task.

A) Left: Example confocal image showing optical fiber track above VTA and expression of ChR2-GFP in midbrain of DAT-Cre mouse. Right: localization of implanted fiber tips from histological slices in all mice.

B) Activation of dopamine neurons during stimulus presentation in the task with reward manipulation. Activation did not change the shifts of psychometric curves.

C) Activation of dopamine neurons during stimulus presentation in a task with no reward manipulation. Optogenetic activations were applied in 40% of randomly chosen trials. The laser pulses started either at the stimulus onset (D,E) or 0.4 s before the stimulus (F,G).

D) Activating dopamine neurons at the time of stimulus onset did not influence current choices (P = 0.96, 1-way ANOVA) or next trials (P = 0.9, 1-way ANOVA) and did not influence visual sensitivity (P = 0.63 signed rank test on the slope of psychometric curves).

E) Activating dopamine neurons at the time of stimulus onset did not influence reaction times (P = 0.67, 1-way ANOVA).

F) Activation of dopamine neurons 0.4 s prior to stimulus presentation did not affect ongoing decisions (P = 0.99, 1-way ANOVA) or subsequent decisions (P = 0.80, 1-way ANOVA).

G) Activating dopamine neurons prior to stimulus onset mildly decreased reaction times (consistent with Hamid et al., 2016), an effect that had low statistical significance in trials with contrast=0 (P=0.05, singed rank test).

H) Left: Suppression or activation of dopamine during the reward time. In consecutive blocks of trials, correct choices to left or right were paired with the laser pulses. Right: The effect of optogenetic suppression.

I) The effect of optogenetic activation.

J) Reaction times separated for stimuli and dopamine activation blocks. Reaction times were smallest for high-contrast stimuli and for response side paired with dopamine.

K) Mean learning curves following switch of dopamine activation side. Shaded regions: mean ± s.e.; curves: model prediction.

L) Performance of an example mouse in experiments in which dopamine neurons were activated in randomly chosen successful trials (~30% of trials). Psychometric functions are plotted as a function of the side chosen on the previous trial, and outcome of that trial. Activation of dopamine neurons in previous trial shifted decisions towards the side paired with such activation, but only when the immediate sensory evidence was weak. Solid curves: predictions of the behavioral model.

M) Estimated model parameters for experiments in which dopamine neurons were suppressed in consecutive blocks of trials at the time of outcome. Circles with different colors represent different animals.

N) Similar to (E) but for the experiments including activation of dopamine neurons in consecutive blocks.

O) Simulation showing V_L and V_R over trials in a model run in which we added a constant (=3) to the value of δ in correct trials of alternating blocks. V_L and V_R stay stable since rewards are contingent to correct sensory detection.
